# Items

**Published:** 1899-05

**Authors:** 


### ITEMS.

AT THE Paris exposition next year an American boarding house, or as they prefer to call it in France, a "pension" is to be opened for the special benefit of medical men and their families who visit the exposition. Professor Wisner and his wife, whose venture it will be, are natives of France, who have been long resident in New York City, and who are well and favorably known to many medical men there. They have taken a mansion near the Bois de Boulogne, and will have it fitted up in such a way as to afford superior accommodation for their guests. A number of prominent American doctors have already engaged apartments from the professor, and it is hoped the house will become the headquarters of the members of the medical profession and their families during their stay in the French capital. Professor Wisner would be pleased to hear from other prospective visitors before leaving for Europe. For the present he may be addressed 605 Madison Avenue, New York City.

---

BUREAU OF THE MEDICAL PRESS.—It has long been a subject of comment that the medical journals were slow to appreciate the advantages and benefits to be derived from a representation at the National and Medical society meetings, and, indeed, exhibiting an indifference in reporting the proceedings. The fact that a half dozen journals are rarely seen at the meetings of the American Medical Association, surely reflects little credit upon the enterprise of our medical publishers. It seems, however, to have an explanation in the matter of expense. Unless the publisher or editor has the leisure time to attend the meeting himself, it is difficult to secure a representative who will do justice to the publication, to say nothing of the expense of sending him and maintaining quarters during the session. Still there is no question that the society meeting affords the very best opportunity for a journal to get in touch with both the profession and the advertiser, and this plan, if systematically followed, will ultimately insure a degree of success unattainable in any other way. We are, therefore, pleased to announce that the bureau service, inaugurated by Mr. Chas. Wood Fassett, several years ago, will be continued at the American Medical Association meeting in Columbus, June 6 to 9, 1899. A catalogue edition of the *American Medical Journalist* will be issued, containing a descriptive index to the medical periodicals and reference books contained in the bureau, and advertising matter of various kinds will be distributed for members.

---

STATE OF NEW YORK CIVIL SERVICE EXAMINATION.—An open competitive examination for the position of medical interne (homeopathic and regular) in the state hospitals, will be held in various cities throughout the state on May 27, 1899. Salary of the position, $600 per annum and maintenance.

Also an examination for assistant in dietary experiments, lunacy commission; salary $100 per month. Intending competitors must file applications in the office of the commission on or before May 22d. For application blank and detailed circular address the secretary, New York Civil Service Commission, Albany, N. Y.

MESSRS. REED AND CARNRICK on April 1, 1899, removed their business from 426-428 West Broadway, N.Y., to their commodious new factory building, Nos. 42, 44 and 46 Germania avenue, Jersey City, N. J. Their post-office address is P. O. Box No. 3042, New York City.

---

THE *Kansas City Medical Index* and *The Kansas City Lancet* has been consolidated under one management. By this arrangement Dr. John Punton becomes editor-in-chief and associated with him are twelve well known physicians and surgeons of Kansas City. In future all correspondence, exchanges, etc., intended for these consolidated journals should be sent to the editor, 600 Altman building, Kansas City, Mo.

---

THE subject of substitution as sometimes practised by dispensers of medicine is one in which every physician is interested. We print the following correspondence as bearing upon the question and which sufficiently explains itself without comment on our part:

CITY OF ST. LOUIS, HEALTH DEPARTMENT.

MAX C. STARKLOFF, M. D., Health Commissioner.

M. J. BREITENBACH CO., 100 Warren street

QUARANTINE P. O., MO., October 30, 1898.

*Dear Sirs*—It has been brought to my notice that . . . . . . are introducing an iron and manganese preparation, and their literature would make it appear that I had expressed a favorable opinion upon their product, by reference made to the *American Therapist*, page 258, 1896. The article in question was in reference to "Gude's Pepto-Mangan," and was not intended to cover all of the so-called iron and manganese preparations, and as this article and clinical report was written solely upon pepto-mangan (Gude), I in a manner hold you responsible for it, and will be greatly obliged if you will take the time and trouble to *deny* the misleading statement of . . . and place me right in the eyes of the profession.

In the article referred to I was very careful to specify Gude's preparation, with this knowledge and object in view when it was written. It is ridiculous to suppose that I would express a favorable opinion upon a preparation, that I positively assure you that I have never seen or heard of. Feeling assured that you will do all in your power to grant my request in this matter,

I remain, yours very truly,

M. C. WOODRUFF, M. D.

